# Genetic characterization of a novel *Salinicola salarius* isolate applied for the bioconversion of agro-industrial wastes into polyhydroxybutyrate

**DOI:** 10.1186/s12934-024-02326-z

**Published:** 2024-02-17

**Authors:** Shymaa A. Abdelrahman, Olfat S. Barakat, Marwa N. Ahmed

**Affiliations:** Department of Microbiology, Faculty of Agriculture, Cairo University, El-Gamaa Street, Giza, 12613 Egypt

**Keywords:** Polyhydroxybutyrate (PHB), FTIR, Bioplastic, *Salinicola salaries*, GC–MS

## Abstract

**Background:**

Polyhydroxybutyrate (PHB) has emerged as a promising eco-friendly alternative to traditional petrochemical-based plastics. In the present study, we isolated and characterized a new strain of *Salinicola salarius,* a halophilic bacterium, from the New Suez Canal in Egypt and characterized exclusively as a potential PHB producer. Further genome analysis of the isolated strain, ES021, was conducted to identify and elucidate the genes involved in PHB production.

**Results:**

Different PHB-producing marine bacteria were isolated from the New Suez Canal and characterized as PHB producers. Among the 17 bacterial isolates, *Salinicola salarius* ES021 strain showed the capability to accumulate the highest amount of PHB. Whole genome analysis was implemented to identify the PHB-related genes in *Salinicola salarius* ES021 strain. Putative genes were identified that can function as *phaCAB* genes to produce PHB in this strain. These genes include *fadA*, *fabG*, and P3W43_16340 (encoding acyl-CoA thioesterase II) for PHB production from glucose. Additionally, *phaJ* and *fadB* were identified as key genes involved in PHB production from fatty acids. Optimization of environmental factors such as shaking rate and incubation temperature, resulted in the highest PHB productivity when growing *Salinicola salarius* ES021 strain at 30°C on a shaker incubator (110 rpm) for 48 h. To maximize PHB production economically, different raw materials i.e., salted whey and sugarcane molasses were examined as cost-effective carbon sources. The PHB productivity increased two-fold (13.34 g/L) when using molasses (5% sucrose) as a fermentation media. This molasses medium was used to upscale PHB production in a 20 L stirred-tank bioreactor yielding a biomass of 25.12 g/L, and PHB of 12.88 g/L. Furthermore, the produced polymer was confirmed as PHB using Fourier-transform infrared spectroscopy (FTIR), gas chromatography-mass spectroscopy (GC–MS), and nuclear magnetic resonance spectroscopy (NMR) analyses.

**Conclusions:**

Herein**,** *Salinicola salarius* ES021 strain was demonstrated as a robust natural producer of PHB from agro-industrial wastes. The detailed genome characterization of the ES021 strain presented in this study identifies potential PHB-related genes. However, further metabolic engineering is warranted to confirm the gene networks required for PHB production in this strain. Overall, this study contributes to the development of sustainable and cost-effective PHB production strategies.

**Supplementary Information:**

The online version contains supplementary material available at 10.1186/s12934-024-02326-z.

## Background

The increasing production of conventional plastics has resulted in the accumulation of tons of plastic waste in the environment, enduring for centuries due to their slow decomposition. These plastics derived from non-renewable resources like fossil fuels [1] have inspired researchers to explore environmentally friendly alternatives. The significance of bioplastics extends beyond their biocompatibility, renewability, non-toxicity, and biodegradability, as they can also be derived from various waste products including cellulose and polysaccharides [2].

Polyhydroxyalkanoates (PHAs) are polyester bioplastics that have garnered substantial attention as promising commercial and green alternatives for fossil fuel plastics due to their safe components and their high biodegradability [3]. PHAs can be fully degraded to carbon dioxide and water via microbial metabolic processes [4]. Polyhydroxybutyrate (PHB) is a short-chain length type of PHA and is formed by the condensation of 3-hydroxybutyric acid [5]. The PHB synthesis operon (*phaCAB*) is involved in the formation of PHB, playing a pivotal role in the conversion of acetyl-CoA to PHB [6]. PHB can be produced by biotechnological processes under controlled conditions. Consequently, the production or degradation of PHB does not impose any adverse impact on the environment.

Various bacterial strains can produce PHB as intracellular particles, like synthetic polymers. PHAs exhibit physico-chemical characteristics including flexibility and toughness [7]. Despite the inherent and environmental advantages of PHB over petrochemical-derived plastics, its industrial production is constrained by the high cost of the involved raw materials, which can constitute up to 40–50% of the total production cost [8].

Advancements in PHB production involve manipulating microbial strains, using mixed microbial cultures, diversifying substrates, and optimizing processes, including scaling up production. Researchers are employing synthetic biology strategies to enhance PHB production [9, 10]. However, these advancements also contribute to increased cost competitiveness, complexities associated with scale-up, and the challenge of ensuring high PHB yields. To overcome these challenges, continuous investigations are essential to identify naturally high PHB-producing strains, low-cost raw materials and optimize fermentation processes.

Investigating industrial or agricultural waste feedstocks as carbon sources for PHB production via microorganisms presents a promising avenue to reduce waste disposable and PHB production costs [11]. Among agro-industrial wastes, salted whey (SW), a major by-product of cheese manufacturing comprising 70–90% of milk content, and molasses, a by-product of the sugar manufacturing process containing approximately 50% (w/w) of total sugars are noteworthy candidates. Sucrose is the main sugar component in sugarcane molasses, which comprises glucose and fructose [12]. Molasses has previously been utilized as a substrate for PHB production by *Bacillus* sp. and *Priestia* sp. [13, 14].

Therefore, the aim of this study was to produce PHB naturally using different isolates of marine bacteria from the New Suez Canal in Egypt and to characterize the genetic pathways involved in PHB production in the most promising PHB-producing isolate. In addition, this study aimed to optimize the production conditions in small flasks and in a 20 L stirred-tank bioreactor and to perform a structural analysis of the produced PHB by FTIR, GC–MS, and NMR spectroscopy.

## Methods

### Isolation of PHB-producing bacteria from seawater

Marine samples were aseptically collected in a sterilized clean bottle from the New Suez Canal, Qantara City, Ismailia, Egypt. Each marine sample was serially diluted and plated into Sucrose/Yeast extract agar medium (SYA) (KH_2_PO_4_ 1 g/L, MgSO_4_ 0.5 g/L, yeast extract 4 g/L, and sucrose 20 g/L) [15] supplemented with 3% NaCl (SYANa) and incubated at 30˚C for 24 h. Thereafter, several bacterial colonies with distinct morphologies were selected and sub-cultured on SYA. The pure bacterial colonies were streaked on SYA slants, incubated at 30°C overnight, and then stored at 4°C for further use.

### Screening the isolated bacteria for PHB production

The isolated bacterial colonies were stained with an alcoholic solution of Sudan Black B (SBB 0.02%). PHB-producing colonies exhibited a bluish-black color while non-PHB-producing colonies exhibited a white color [16]. Bacterial smears were prepared from the pure isolates grown on SYANa plates. Smears were stained with Sudan Black-B (SBB) solution for 10 min [17], followed by counterstaining with 0.5% aqueous safranine for 5s. Subsequently, the slides were examined under the light microscope (Leica MZ 16) (100x). Additionally, culture plates were subjected to SBB staining for 30 min, followed by light microscope examination.

Nile Blue-A stain was also used to stain bacterial smears prepared from the isolated strains according to the method described by Ostle and Holt [18]. The stained smears were examined under the fluorescence microscope (Leica CH 9435 Herrburg) at a wavelength of 490 nm. PHB granule-producing bacterial isolates exhibited a bright yellowish-orange fluorescence under the microscope. The PHB-producing isolates were selected and stored at -20°C for further use.

### Submerged fermentation for PHB production

The PHB production was conducted in 250 ml Erlenmeyer flasks, each containing 100 ml of sterilized Sucrose/Yeast extract broth medium (SYB) supplemented with 3% NaCl (SYBNa). The flasks were inoculated with 1 ml of the bacterial inoculum (17 isolates) and then incubated at 25°C for 48 h. To assess the effect of temperature and shaking rate on the PHB production, the flasks were incubated at 25 and 30°C under both static and shaking conditions (110 rpm, Biobock Saewfc, France) for 48 h, using the selected isolate (ES021).

The dry cell weight (biomass, g/L) and PHB concentration (g/L) were determined at the end of the fermentation process to estimate the percentage of PHB yield.

### Whole bacterial genome sequencing of the most promising PHB-producing isolate

Genomic DNA was extracted from the isolate ES021 that showed the highest levels of PHB production using Gentra puregene yeast/bacteria DNA purification kit (Qiagen, cat. no. 158567) according to the manufacturer’s instructions. The DNA libraries were prepared using Illumina Truseq DNA nano kit (cat no. 20015964) and sequenced on an Illumina Miseq with a coverage of approximately 50x. The quality of paired end sequencing reads was checked using FastQC tool [19]. The reads were then de-novo assembled using Unicycler (v0.4.8). QUAST tool (v5.0.2) was used to check the quality of genome assembly. The genome completeness of the assembled contigs was comprehensively assessed using Benchmarking Universal Single-Copy Orthologue (BUSCO v5.5.0) database bacteria and *Gammaprotebacteria* _0db10 [20]. The annotation of the assembled genome contigs was performed using RAST tool kit (RASTtk) [21]. Functional annotation was conducted using PATRIC, the all-bacterial Bioinformatics Database and Analysis Resource Center on-line platform (www.bv-brc.org) and KEGG pathway database. For publication, the NCBI Prokaryotic Genome Annotation Pipeline was used later for the genome annotation [22]. The genome features were visualized through a circus plot using the Proksee platform (https://proksee.ca) [23].

For comparative genomics, the genetic distance between the genome sequence of ES021 strain and genomic sequences of closely related bacteria was calculated using Mash/MinHash provided by PATRIC genomic comparative analysis tool.

Phylogenetic analysis was performed using Molecular Evolutionary Genetics Analysis Version 11 (MEGA 11) to align the 16S rRNA sequences employing Muscle algorithm (Gap open: -400, gap extend: 0, and UPGMA clustering) [24]. Subsequently, the Maximum Likelihood (ML) method was utilized for constructing the 16S rRNA tree. Evolutionary distances were computed using the Kimura 2-parameter method [25], and the phylogeny was tested through 1000 bootstrap replicates. The resulting tree was visualized using Itol v6 [26]. The average nucleotide identity based on blast algorithm (ANIb) in JSpecies web server (www.ribocon.com) was implemented to align the whole genome sequences and generate an ANI matrix. Heatmap.2 function in R v4.3.2 was used to construct a dendrogram.

### Optimization of PHB production process parameters using agro-industrial wastes

Optimization of the fermentation process parameters for maximum PHB production on SW and molasses using the bacterial strain *Salinicola salarius* ES021 was conducted in batches in 250 ml Erlenmeyer flask containing the fermentation medium (100 ml working volume). The effect of fermentation factors: sugar concentration, NaCl concentration, temperature, pH, shaking rate, inoculum size, and incubation period were investigated. The inoculum was prepared using SYBNa.

### Raw materials

Salted whey (SW) containing 2% either, 4% salt, and 5% sugar, was obtained from Dairy Department, Faculty of Agriculture, Cairo University, Giza, Egypt. SW was clarified according to Gupte and Nair [27].

Sugarcane molasses containing 40.5% sugar, was obtained from Sugar and Integrated Industries Company, Hawamdia, Giza, Egypt. Molasses was clarified according to Panda et al. [28].

### Effect of fermentation media on PHB production

Different media formulations were prepared from SW to study the impact of salt, sugar concentrations, and yeast extract (4 g/L) supplementation as well as mineral salts (KH_2_PO_4_ 1 g/L and MgSO_4_ 0.5 g/L) on PHB production as shown in Table 1. In M1 and M2 media, sugar and salt concentrations were maintained at 4 and 5%, respectively, with or without yeast extract and minerals. In M3 and M4, the salt concentration was adjusted to 3%, mirroring the salt concentration in SYBNA with and without yeast extract and minerals. M5 and M6 featured a reduced salt concentration of 2% to explore the impact of lower salt levels on PHB production. Constant sugar concentrations were maintained across most media formulations, except in M7 and M8, where sugar concentrations were decreased to 2% to assess their effect on PHB production. The fermentation media were prepared in 250 ml Erlenmeyer flasks, each containing 100 ml of the sterilized SW media, and inoculated with 1% inoculum from the 48-h-old culture of ES021 strain. The flasks were incubated at 30°C for 48 h. PHB concentration, bacterial dry cell weight (DCW), and PHB yield % were measured at the end of fermentation.

**Table 1:** Fermentation media formulated from SW and their constituents used for optimization of PHB production via *Salinicola salarius* ES021 strain

Media no.	NaCl concentration (%)	Sugar concentration (%)	Yeast extract	Mineral salts
M1	4%	5%	−	−
M2	4%	5%	+	+
M3	3%	5%	−	−
M4	3%	5%	+	+
M5	2%	5%	−	−
M6	2%	5%	+	+
M7	3%	2%	−	−
M8	3%	2%	+	+

### Effect of incubation temperature on PHB production

Fermentation medium M4 supplemented with 3% sugar and 5% salt concentration was incubated at five distinct temperatures (25, 27.5, 30, 32.5, and 35°C). The PHB production was estimated following a 48-h growth period of the ES021 strain.

### pH values

The pH values of the fermentation medium M4 were adjusted at 6.2, 6.5, 7, 7.3, and 7.6. All flasks were inoculated with a 1% inoculum of the ES021 strain and incubated on a shaking incubator (110 rpm) for 48 h at 30°C. Thereafter, PHB yield % was quantified.

### Inoculum size

To assess the impact of inoculum size on PHB production, different inoculum sizes (0.5, 1, 1.5, 2, 2.5, and 3 % v/v) were supplemented to the fermentation medium M4 (pH = 7). The flasks were incubated at 30°C with continuous shaking at 110 rpm for 48 h.

### Shaking rate

To determine the effect of shaking rate, liquid medium M4 (pH = 7) was inoculated with 1% (inoculum size v/v) and incubated at 30°C under static conditions as well as with shaking at 110, 130, 150, and 190 rpm for 48 h.

### Incubation period

The impact of incubation duration on the PHB production was assessed by inoculating the liquid medium M4 (pH = 7) with a 1% inoculum (v/v). The flasks were then incubated at 30 °C with shaking (150 rpm) for varying incubation periods, including 12, 24, 36, 48, 60, 72, 84, and 96 h.

### Production of PHB from molasses and mixture of SW and molasses

Molasses was clarified to achieve sucrose concentration of 5 and 10% as a carbon source and supplemented with 3% NaCl to be utilized for PHB production using the ES021 strain. Furthermore, a combination of SW M4 medium and molasses were used as a fermentation medium for PHB production via the ES021 strain in a ratio of 1:1 and 2:1, resulting in sugar concentrations of 10 and 15%, respectively. All batches were carried out in conical flasks, each containing 100 ml volume, maintained at 30°C with shaking (150 rpm) for 48 h and an inoculum size of 1% (v/v).

### Scaling up PHB production in a 20 L bioreactor:

To enhance the biomass and PHB accumulation by *Salinicola salarius* ES021 strain, batch cultivation was carried out in a 20 L stirred-tank bioreactor containing 15 L of optimized media (molasses 5% sugar, 3% NaCl, KH_2_PO_4_ 1 g/L, yeast extract 4 g/L, and MgSO_4_ 0.50 g/L) inoculated with a 1% of bacterial inoculum and maintained for a duration of 48 h. The operating conditions, including temperature, pH, aeration, and agitation speed were maintained at 30°C, 7.0, 2.5 vvm, and 150 rpm, respectively.

### Determination of PHB dry weight, dry cell weight, and PHB yield

The concentration (dry weight) of extracted PHB was determined as g/L using the method described by Williamson and Willinson, as well as Hahn et al. [29, 30]. The total biomass dry cell weight (DCW) was estimated as g/L according to the method described by Kunioka and Nakamura as well as Hawas et al. [31, 32]. The efficiency of PHB production via the bacterial strain was expressed as PHB yield (%) and calculated as the ratio of PHB concentration (dry weight, g/L) to DCW (g/L), multiplied by 100.

### Biopolymer chemical analysis

The structure of extracted PHB was analyzed using FTIR and GC-MS, and the produced spectrum was compared with the PHB standard (Sigma cat no: 29435-48-1). NICOLET 380 FTIR (Thermo Scientific, China) was applied to identify the functional groups in the produced polymer [33]. To determine the chemical composition of the produced polymer, GC-TSQ mass spectrometer (Thermo Scientific, USA) was used [34]. NMR analysis of the extracted PHB was performed according to the method described by Kanzariya et al. [35]. ^1^H NMR spectra were recorded on a Varian Mercury VX-300 NMR spectrometer. H1 spectra were run at 300 MHz in deuterated dimethylsulphoxide (DMSO-d6).

### Statistical analysis

All experiments were conducted in triplicates, and all data were recorded as means of three replicates. The factors were considered significant if the *P* value is ≤ 0.05 using analysis of variance (ANOVA). Duncan’s multiple range test was used to test the significance of variance treatments (P < 0.05). The statistical analysis and figures were conducted using GraphPad Prism 7, Python v.3.9, and R v4.3.2.

## Results and discussion

### Isolation and screening of PHB- producing bacteria

**Fig. 1:**
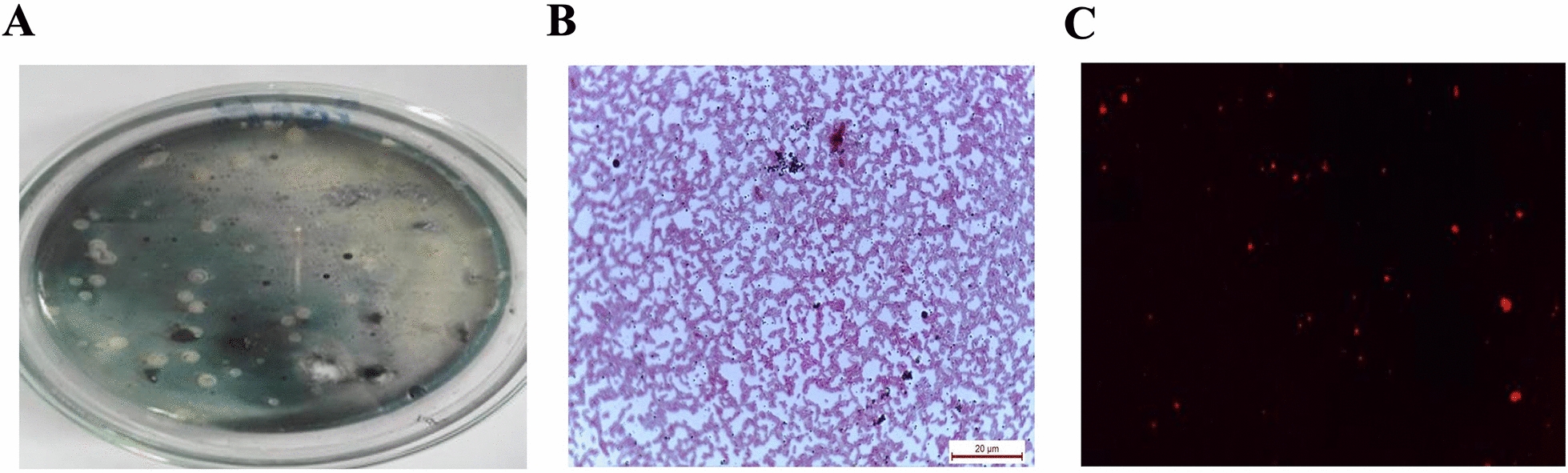
Photomicrograph of the isolate ES021 showing the produced PHB granules inside the cells **A** Rapid screening of PHB production by Sudan Black-B staining (culture staining) showing black spots indicative of PHB granules. **B** Sudan Black-B staining of a bacterial smear (slide staining) showing PHB granules (black spots) surrounding the cells. **C** Nile blue A staining of a bacterial smear (slide staining)

Several bacterial isolates were isolated from the New Suez Canal, in Egypt. A total number of 36 bacterial isolates were screened for PHB production using both rapid-plate screening (Fig. 1A) and Sudan Black B staining of bacterial cells. Among the screened isolates, only 17 isolates were found to be PHB producers based on rapid-plate screening and Sudan Black B staining. Microscopic analysis revealed the presence of lipophilic black granules in stained bacterial cells (Fig. 1B), confirming PHB granule production. The isolates were further purified, Gram-stained, and examined using a light microscope (100x). Most isolates belonged to the genus *Bacillus* (15), with two identified as Gram-negative short rods. Confirmation of PHB production was achieved through Nile blue A staining, followed by fluorescence microscope examination (Fig. 1C). Sudan Black B and Nile blue A are lipophilic dyes characterized by their high selective binding to lipid-rich structures including PHB, allowing their visualization under microscope [36–40].

### Submerged fermentation for PHB production

**Fig. 2:**
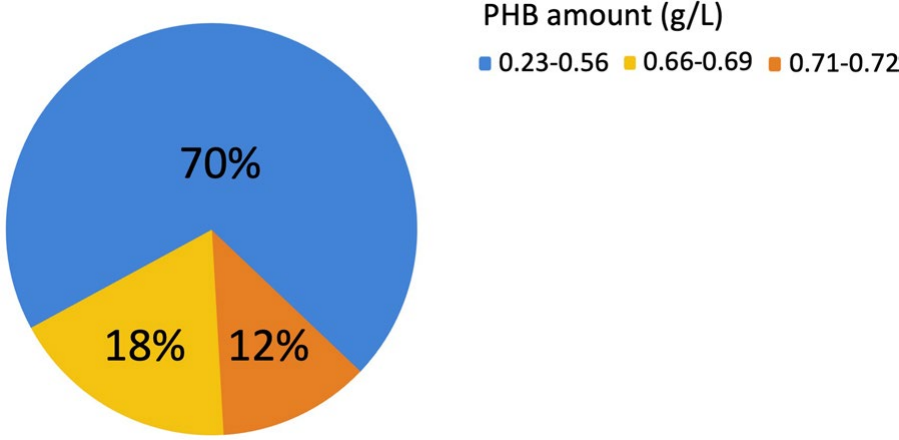
The percentage of bacterial isolates that produce different PHB quantities ranging from 0.23 to 0.72 g/L

The batch culture technique was used to evaluate the PHB production in the selected 17 isolates. Figure 2 shows the percentage of isolates that produced different amounts of PHB.

The results revealed that the two short-rods G-negative isolates produced the highest amount of PHB (0.71-0.72 g/L). Consequently, the isolate ES021 was selected to enhance PHB production in SYBNa medium by investigating the influence of incubation temperature (25 and 30°C) and shaking rate (static and 110 rpm) on the PHB productivity. At the end of the experiment, PHB concentration (g/L), DCW, and PHB yield % were estimated.

**Fig. 3:**
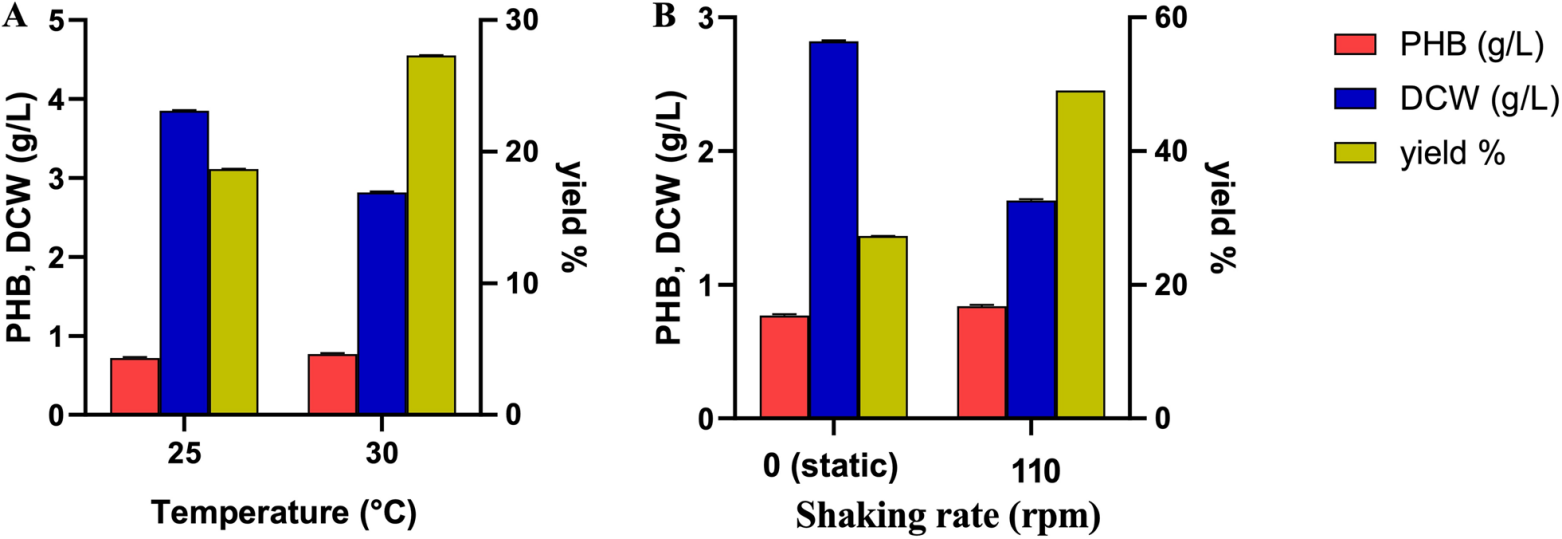
The effect of incubation temperature (**A**) and shaking rate (**B**) on the PHB productivity by the isolate ES021

The incubation temperature was shown to affect the PHB production, with the the PHB amount (g/L) increasing from 0.72 to 0.77 g/L when the incubation temperature was raised from 25 to 30°C (Fig. 3A). Notably, incubation at 25°C favored biomass production, while incubation at 30°C was more promotive to PHB production, resulting in a 6.94% increase. Therefore, the PHB yield was enhanced when growing the isolate ES021 at 30°C. Consistently**,** previous studies reported PHB production by various species of G-bacteria is temperature dependent. Hawas *et al* reported a higher amount of PHB production by *Pseudomonas* sp*.* at 30°C (3.5 g/L) compared to 25°C (0.600 g/L) [32], Mostafa *et al* used *Pseudodonghicola xiamenensis* to accumulate the highest amount of PHB** (**15.54 g/L) at 30°C [2].

Furthermore, growing the isolate ES021 under shaking conditions at 110 rpm demonstrated an improvement in PHB productivity. The PHB amount increased from 0.77 to 0.80 g/L at 30°C under shaking conditions (110 rpm) compared to static conditions. These findings highlight the importance of shaking for enhanced PHB production. Previous studies have shown that PHB yield significantly increased when growing *Bacillus* sp. at 37°C for 48 h under shaking at 150 rpm [37]. Similarly, PHB production by *Bacillus mycoides* DFC1 strain was influenced by incubation temperature and shaking rate, resulting in increased PHB amount (1.8 g/L) and biomass (3.2 g/L) with a yield of 57.20 % under incubation temperature at 37°C and shaking at 120 rpm for 48 h [41].

### Genome characterization

**Fig. 4:**
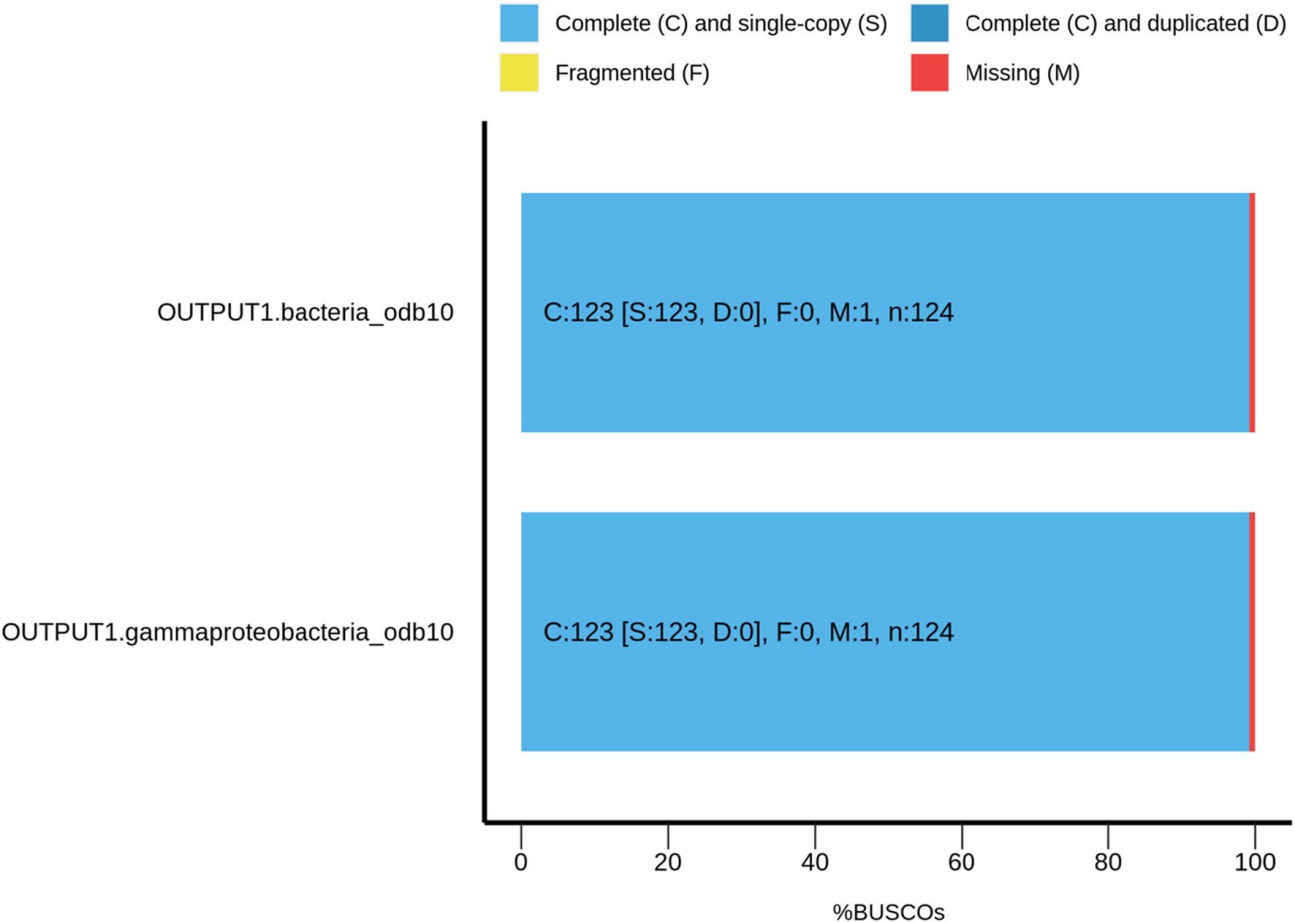
BUSCO assessment of the genome assembly completeness using the bacteria and *Gammaprotebacteria*_odb10 database. Single-copy orthologs (123, 99.2%) were found in the *Salinicola salarius* ES021 genome matched with 124 orthologs in bacteria and *Gammaprotebacteria* database. Only one missing gene was identifed in the *Salinicola salarius* ES021 genome

To reveal the genetic features and identify the pathways involved in PHB production, whole genome sequencing of the isolate ES021 was conducted at 50× coverage. The whole genome was assembled using Unicycler into 24 contigs with a genome length of 3,823,256 bp and an average GC content of 62.67%. The N50 length (the shortest sequence length at 50% of the genome) is 382,245 bp, and the L50 count (the smallest number of contigs whose length sum produces N50 is 4). The completeness of genome assembly was evaluated using BUSCO database specific to bacteria and *Gammaprotebacteria* database, which encompasses 4085 genomes. BUSCO assessment revealed 123 complete single-copy orthologs, accounting for 99.2% completeness and only one missing in the *Salinicola salarius* ES021 strain (0.8%) (Fig. 4). These outcomes establish the high-quality nature of the *Salinicola salarius* ES021 genome, rendering it well suited for the precise identification of PHA and PHB synthesis genes.

The isolate ES021 was identified using the NCBI blast tool as *Salinicola salarius* and deposited in DDBJ/ENA/GenBank under the accession JARJLD000000000. The genome of *Salinicola salarius* was annotated using RASTtk applying the genetic code 11. This bacterium belongs to the *Halomonadaceae* family. This genome has 3530 protein-coding sequences (CDS), 60 transfer RNA (tRNA) genes, and 4 ribosomal RNA (rRNA) genes. The genome annotation included 926 hypothetical proteins and 2604 proteins with functional assignments. The proteins with functional assignments included 979 proteins with Enzyme Commission (EC) numbers, 823 with Gene Ontology (GO) assignments, and 741 proteins that were mapped to KEGG pathways.

**Fig. 5:**
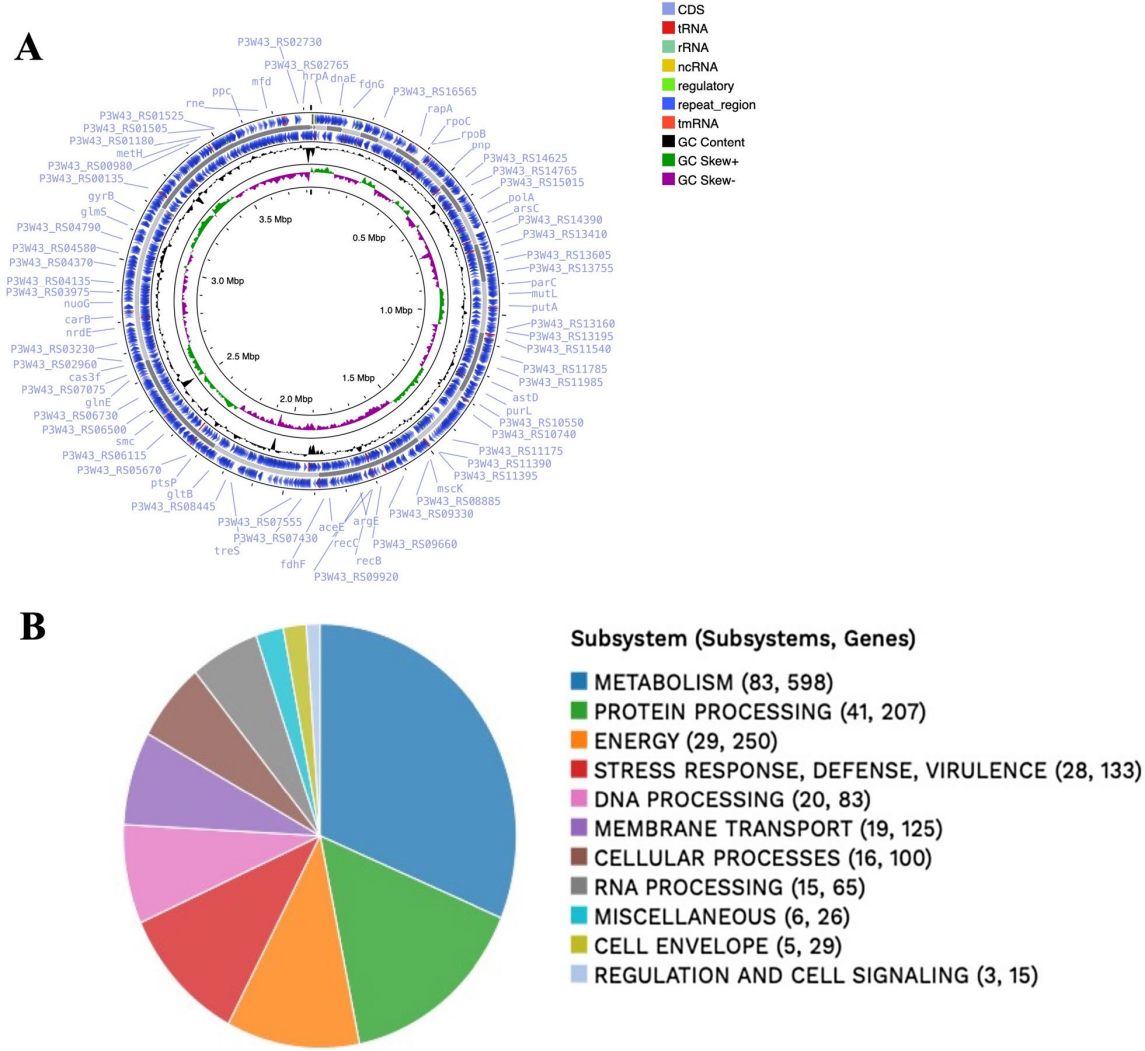
Genetic distribution of the genetic features and subsystems in the genome of *Salinicola salarius* strain ES021. **A** Circular graphical distribution of the genome annotation showing RNA genes, GC content, and GC skew. **B** An overview of the subsystems detected in this genome showing the number of subsystems and number of genes involved

A circos plot of the genome annotation distributions is shown in Fig. 5A. This shows from outer to inner rings, the contigs, CDS on the forward strand, CDS on the reverse strand, RNA genes, GC content, and GC skew. The identified subsystems and the number of corresponding genes within each subsystem are shown in Fig. 5B, providing insights into the functional categories of the genome.

The annotated genes have homology to known transporters, virulence factors, drug targets, and antibiotic resistance genes. The specialty genes and the specific source database where homology was found are shown in (Table 2). The list of annotated genes, their functions, sequences, and locations are shown in Additional file 2: Data Set 1.

**Table 2:** List of specialty genes in the genome of *Salinicola salarius* ES021 strain

Specialty genes	Genes number	Source
Antibiotic resistance	29	PATRIC
Drug target	7	Drug Bank
Transporter	2	TCDB
Virulence factor	4	PATRIC-VF

**Fig. 6:**
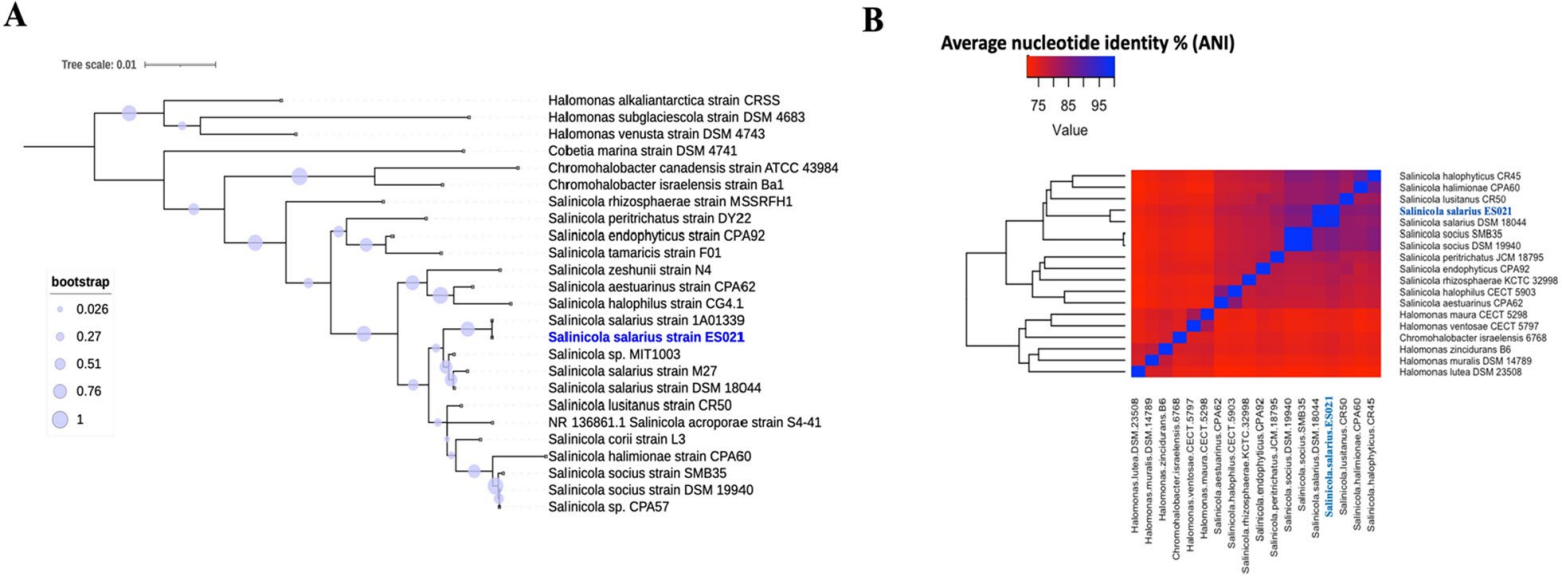
Phylogenetic analysis of *Salinicola salarius* ES021 strain in relation to other taxonomic groups based on the 16S rRNA gene and whole genome sequences. **A** Maximum Likelihood (ML) tree based on 16S rRNA gene analysis depicts the phylogenetic relationships among closely related species. Bootstrap values exceeding 75% (based on 1000 replicates) are represented as bubbles on the tree. **B** The Average Nucleotide Identity (ANI) dendrogram of whole genome sequences. ANI similarity values are color-coded, ranging from 75% (shown in red) to 100% (shown in blue)

Phylogenetic analysis of the 16S rRNA genes from *Salinicola*, *Halomonas*, and *Chromohalobacter* strains, conducted using the Maximum Likelihood (ML) algorithm, elucidated the phylogenetic relationships among these genera. As shown in Fig. 6A, the analysis revealed the placement of *Salinicola salarius* ES021 strain in relation to closely related strains within these genera.

*Salinicola salarius* ES021 strain exhibited the highest similarity to the 16S rRNA gene of *Salinicola salarius* 1A01339 strain, demonstrating a close phylogenetic relationship between these two strains.

The alignment of whole genome sequences and determination of ANI% can enhance depth and robustness of phylogenetic analysis compared to relying solely on the 16S rRNA gene-based phylogenetic analysis. The complete genome sequence of *Salinicola salarius* ES021 strain was aligned with 18 genomes belonging to *Salinicola*, *Halomonas,* and *Chromohalobacter* strains. ANI analysis showed that *Salinicola salarius* ES021 strain is closely related to *Salinicola salarius* DSM 18044 (ANI > 97%) (Fig. 6B). Furthermore, *Salinicola salarius* ES021 strain showed a close genetic relationship to *Salinicola socius* strains (ANI > 85%).

Therefore, the implementation of 16S rRNA and ANI analyses consistently supports the taxonomic placement of *Salinicola salarius* ES021 strain as a strain of *Salinicola salarius*.

Mash/MinHash was used to estimate the genetic distance between *Salinicola salarius* ES021 strain and other sequenced *Salinicola* species (Additional file 1). The genetic distance between ES021 strain and *Salincola salarius* DSM 18044 strain is 0.01 showing a significant difference between both strains.

**Fig. 7:**
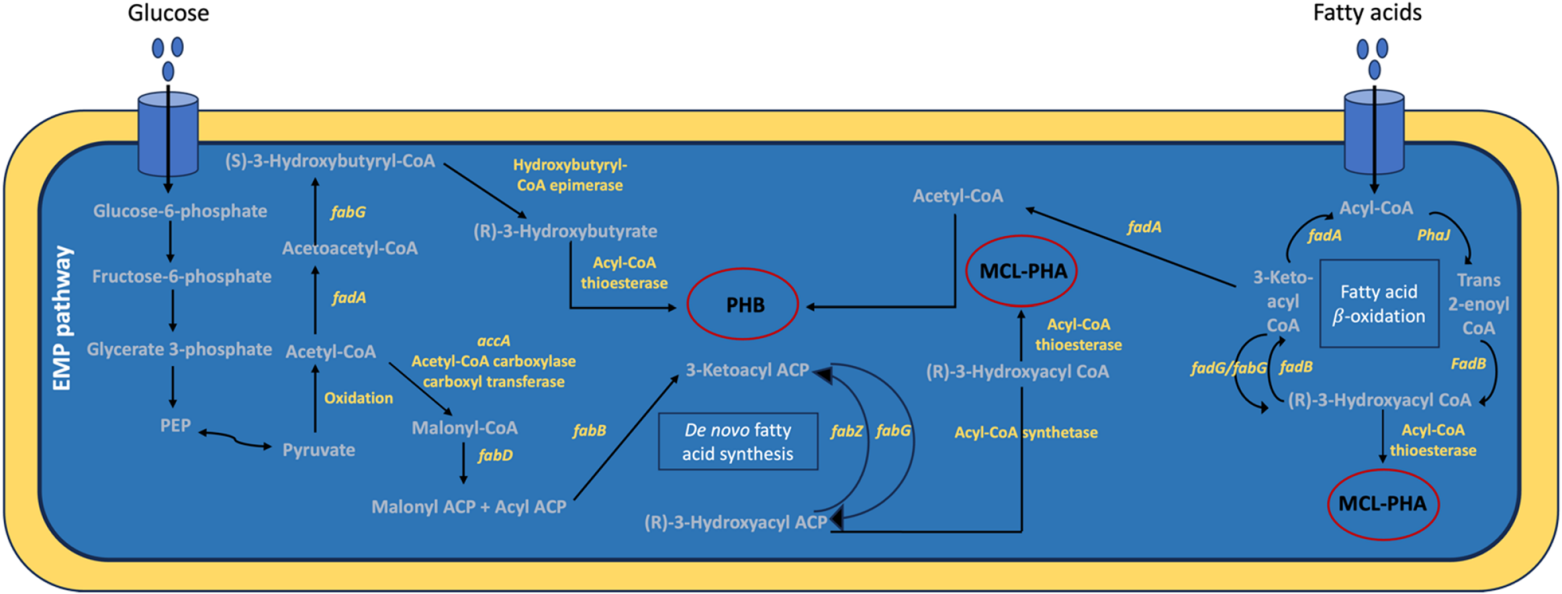
Overview of the PHB and MCL-PHA synthetic pathways in *Salinicola salarius* ES021 strain through EMP pathway, De novo fatty acid synthesis pathway, and fatty acid β-oxidation pathway

The genetic pathways for PHB and medium-chain-length PHA (MCL-PHA) production in *Salinicola salarius* ES021 strain were analyzed and predicted (Fig. 6). Interestingly, the PHA synthesis operon *phaCAB* does not exist in the ES021 strain. In most bacteria, PHB production proceeds in three steps mediated by the genes located in the *phaCAB* operon [42, 43]. These genes encode three enzymes including β-ketothiolase (PhaA), acetoacetyl-CoA reductase (PhaB), and PHA synthase (PhaC) [44, 45]. However, alternative genes that can function as *phaCAB* genes were predicted in *Salinicola salarius* strain ES021 leading to the construction of distinct PHB and MCL-PHA synthetic pathways (Fig. 7). List of putative genes that were predicted to be involved in the PHB and MCL-PHA production is shown in Table 5.

Acetyl CoA is the precursor of PHB synthesis, which is produced through the oxidation of pyruvate by the pyruvate dehydrogenase enzyme. Pyruvate is mainly produced through glucose metabolism via the Embden-Meyerhof-Parnas (EMP) pathway, pentose phosphate (PP) pathway, and Entner-Doudoroff (ED) pathway. The three pathways are detected in this ES021 strain using the KEGG pathway. Additionally, the genome of ES021 strain was found to harbor genes encoding enzymes involved in sucrose and starch metabolism. These enzymes include UTP-glucose-1-phosphate uridylyltransferase, glucokinase and alpha-amylase. Both UTP-glucose-1-phosphate uridylyltransferase and glucokinase play pivotal role in sucrose synthesis and degradation, while alpha-amylase is related to starch metabolism.

For PHB production from glucose in the ES021 strain, the enzyme 3-ketoacyl-CoA thiolase encoded by the *fadA* gene was speculated to be involved in the condensation of two acetyl-CoA molecules into acetoacetyl-CoA. 3-ketoacyl-CoA thiolase has been reported previously to catalyze the condensation reaction between two acyl-CoAs and acetyl-CoAs [46]. *fadA* gene was identified as a second copy of *phaA* gene in the halophilic marine bacterium *Cobetia* sp. MC34 [47]. The amino acid sequence of this gene has 95.7% similarity to the *phaA* gene in *Cobetia marina* DSM 4741^T^ strain and 42.4% to the *phaA* gene in *Halomonas* sp. SF2003 [48].

The reduction of acetoacetyl-CoA to (*S*)-3-hydroxybutyryl-CoA in the ES021 strain is catalyzed by *fabG* that can function as *phaB* encoding 3-oxoacyl-ACP reductase. In the cyanobacterium *Synechocystis* sp., FabG has been shown to have the same function as PhaB and can replace PhaB as an acetoacetyl-CoA reductase for PHB production [49, 50]. *In vivo* studies showed that FabG in *Pseudomonas aeruginosa* can work as β-ketoacyl-CoA reductase for PHB production in *E. coli* [51]. Furthermore, the structures of both FabG and PhaB from *Synechocystis* sp. were shown to be very similar [52]. This indicates that FabG could act as an acetoacetyl-CoA reductase (phaB) in *Salinicola salarius* ES021 strain to produce PHB.

The (*S*)-3-hydroxybutyryl-CoA is converted into (*R*)-3-hydroxybutyryl-CoA by hydroxybutyryl-CoA epimerase enzyme. The conversion of 3-hydroxybutyryl-CoA into (*R*)-3-hydroxybutyrate was predicted in this study to be mediated by acyl-CoA thioesterase II that can function as PHB synthase (*phaC*) catalyzing the removal of CoA-SH from 3HB. PHB synthase is the key enzyme for PHB synthesis. It catalyzes the condensation of PHB monomers to PHB [53, 54]. In *E. coli*, thioesterase II was reported to be capable of hydrolyzing 3-hydroxybutyryl-CoA turning it into 3-hydroxybutyrate, which is the monomer of PHB [55, 56].

For PHB and MCL-PHA production from fatty acids, the acetyl-CoA is converted into malonyl-CoA by acetyl-CoA carboxylase carbonyl transferase to enter the De novo fatty acid synthesis pathway. A cascade of metabolic reactions is mediated in this pathway through the action of enzymes encoded by *fadBDG* genes (Fig. 6). In the fatty acid β-oxidation pathway, acyl-CoA acts as a precursor to produce both MCL-PHA and PHB. Acyl-CoA can be converted into acetyl-CoA through the action of enzymes encoded by *phaJ* and *fadAB* genes. The produced acetyl-CoA can mediate the production of PHB as aforementioned. MCL-PHA can be produced from acyl-CoA by converting it into 3-hydroxyacyl-CoA. Acyl-CoA thioesterase II enzyme can convert 3-hydroxyacyl-CoA into PHA through the removal of CoA-SH (Table 3).

**Table 3:** Putative PHB and MCL-PHA-related genes in the genome of *Salincola salarius* ES021 strain

Gene	Function	Gene number	Protein_accession
FadA	3-Ketoacyl-CoA thiolase/ Acetyl-CoA acetyltransferase	3	MDF3919929.1
FadB	Fatty acid oxidation complex subunit alpha	1	MDF3919930.1
phaJ	Enoyl-CoA hydratase	5	MDF3918188.1
FabA	3-Hydroxyacyl-[acyl-carrier-protein] dehydratase	2	MDF3920313.1
FabB	Beta-ketoacyl-ACP synthase I	1	MDF3920314.1
FabD	ACP S-malonyltransferase	1	MDF3917661.1
FabF	Beta-ketoacyl-ACP synthase II	2	MDF3917658.1
FabG	3-Oxoacyl-ACP reductase	4	MDF3917660.1
P3W43_16340	Acyl-CoA thioesterase II	1	MDF3919661.1

### Fermentation media

Salted whey (SW) served as the fermentation medium for PHB production, with different formulations tested involving different sugar and salt concentrations. Yeast extract and minerals were introduced into the salted whey to investigate their impact on PHB productivity. Table 4 presents the PHB quantities (g/L), dry cell weight (DCW, g/L), and PHB yield (%) by the ES021 strain under different SW medium conditions. The results reveal that the PHB production capacity of the ES021 strain is influenced by sugar concentration, salt (NaCl) levels, and the addition of yeast extract and minerals to the SW medium. Elevated salt concentrations exhibited a notable effect on PHB production, with a decrease of over 9% observed in M1 when salt concentration increased from 2 to 4%. Notably, the highest PHB production (3.46 g/L) was achieved in the M4 culture medium containing 5% lactose, supplemented with yeast extract and mineral salts (Table 4). Further investigation into the effect of sugar levels indicated that increasing lactose quantity in the fermentation medium stimulated PHB production. The optimal level for PHB production was found to be 5% lactose, resulting in a PHB amount of 2.56 g/L, with DCW reaching 4.56 g/L and a PHB yield of 56.14%. Thus, fermentation media containing 2% and 5% lactose levels were applied to study the influence of yeast extract and mineral salt supplementation on PHB productivity. The addition of yeast extract and mineral salt significantly influenced the conversion of lactose into PHB, irrespective of sugar or salt concentration. The highest PHB amount (3.46 g/L) was achieved when yeast extract and mineral salts were added to the SW medium containing 5% lactose and 3% NaCl (M4). Therefore, M4 was selected to study the impact of other growth parameters on PHB production. Whey from cheese, produced globally in substantial quantities, is a promising raw material for PHB production. The ES021 strain demonstrated the ability to accumulate a significant amount of PHB from lactose and SW, highlighting its potential in utilizing whey as a cost-effective substrate for PHB manufacturing. Numerous studies have documented PHB production from whey by indigenous microorganisms. Elmashi et al. and Szacherska et al. reported PHB production from whey using *Bacillus cereus* and *Pseudomonas* sp*.,* respectively [57, 58]. Whey also necessitates a costly treatment prior to disposal, making it an attractive raw material for PHB manufacturing. ES021 strain has demonstrated a high capability to accumulate a significant amount of PHB from lactose and SW.

**Table 4:** Effect of different fermentation media on PHB production using the ES021 strain

Media	Salt conc	Sugar conc.	PHB g/L	DCW g/L	PHB yield
M1	4%	5%	1.09 ± 0.05	6.78 ± 0.3	16.08
M2^+^	4%	5%	1.76 ± 0.01	6.26 ± 0.4	28.12
M3	3%	5%	2.56 ± 0.01	4.56 ± 0.2	56.14
M4^+^	3%	5%	3.46 ± 0.02^****^	5.96 ± 0.1	58.05
M5	2%	5%	1.30 ± 0.06	2.76 ± 0.01	47.10
M6^+^	2%	5%	1.93 ± 0.03	3.12 ± 0.02	61.86
M7	3%	2%	0.84 ± 0.01	1.86 ± 0.01	45.16
M8^+^	3%	2%	1.03 ± 0.01	1.21 ± 0.06	85.12
LSD 0.05			0.0890	0.5412	

^**+**^Addition of yeast extract and mineral salts, *P* value < 0.0001

Incubation temperature was 30°C for 48 h, inoculum size=1%, and pH=6.2+ 0.2. Data are represented as means of three independent replicates ± standard error of the mean

### Incubation temperature

**Fig. 8:**
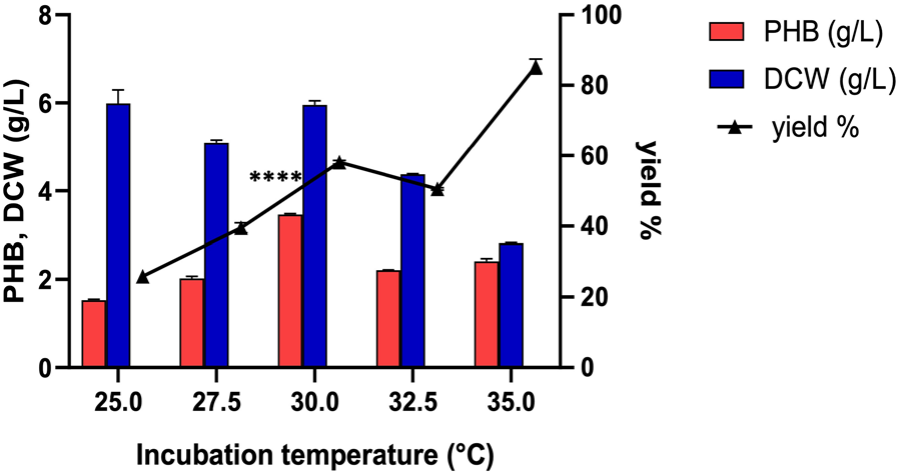
Effect of different incubation temperatures on PHB production by the ES021 strain. The Incubation period was for 48 h under shaking at 110 rpm with an inoculum size of 1% in the fermentation medium M4. **** *P* < 0.0001. Data are presented as values of three independent replicates. The error bars represent the standard error of the mean

The ES021 strain was allowed to grow at five different temperatures (25, 27.5, 30, 32.5, and 35°C) to optimize PHB production. Given that the optimal temperature for *Salincola salarius* growth is 30°C, this temperature, along with two points below and higher, was selected for further PHB production assessment. Fig. 8 and Additional file 3: Table S1 illustrate that the incubation of the ES021 strain at 30°C resulted in the highest PHB production, reaching 3.46 g/L after 48 hours at pH=6.2. Lower yields were observed at 25°C, and the difference in PHB production at 30°C compared to 25°C was statistically significant (P < 0.0001). This finding aligns with previous studies demonstrating the significant impact of incubation temperature on PHB production [12, 59, 60]. For instance, Mostafa et al. achieved the highest PHB production by cultivating *P. xiamenensis* at 35°C for 96 hours [2].

### pH value

**Fig. 9:**
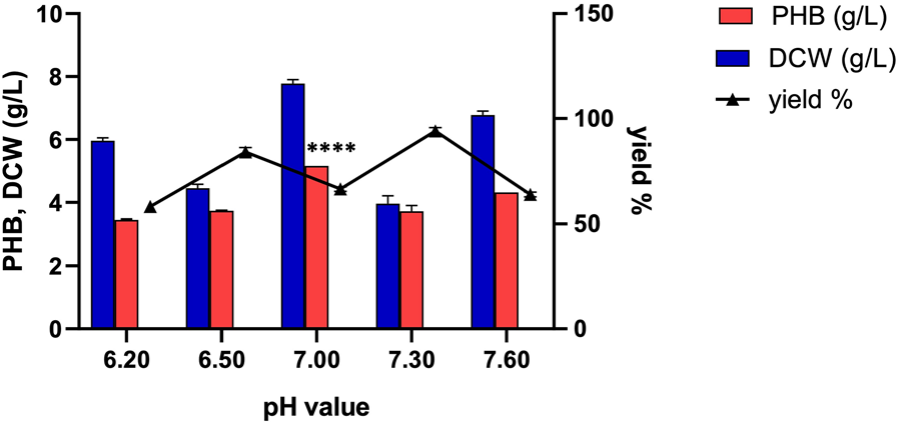
Effect of different pH values on PHB production by the ES021 strain. Incubation was at 30°C for 48 h under shaking at 110 rpm with an inoculum size of 1% in the fermentation medium M4. ****P < 0.0001. Data are presented as values of three independent replicates. The error bars represent the standard error of the mean

The ES021 strain demonstrated the highest PHB production at 30°C and pH 7, yielding 5.16 g/L of PHB with a corresponding PHB yield of 66.32% after 48 h (Fig. 9 and Additional file 3: Table S2). This finding agrees with previous studies suggesting that a pH of 7.5 is optimal for PHB production [61]. Additionally, studies on *Rhizobium* strains cultured in yeast extract mannitol broth indicated an increase in PHB production with the elevation of pH to 7 (from 0.01 to 0.5 g/L culture) [62].

### Inoculum size

**Fig. 10:**
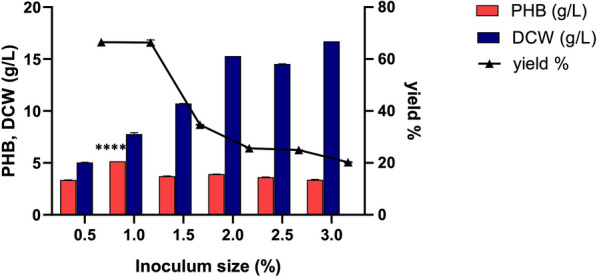
Effect of different inoculum sizes on PHB production by the ES021 strain. Incubation was at 30°C for 48 h under shaking at 110 rpm at pH = 7 in the fermentation medium M4. **** *P* < 0.0001. Data are presented as values of three independent replicates. The error bars represent the standard error of the mean

Various inoculum sizes (0.5, 1, 1.5, 2, and 2.5%) were examined to determine the optimum size for maximizing PHB production. As depicted in Fig. 10 and Additional file 3: Table S3, the ES021 strain exhibited significantly higher PHB levels (5.16 g/L, P < 0.0001) at an inoculum size of 1% compared to other sizes. Abdel Kareem et al. showed that increasing the inoculum size to 4 and 5% resulted in lower PHB amounts, when using whey as a fermentation medium [34].

### Shaking rate

**Fig. 11:**
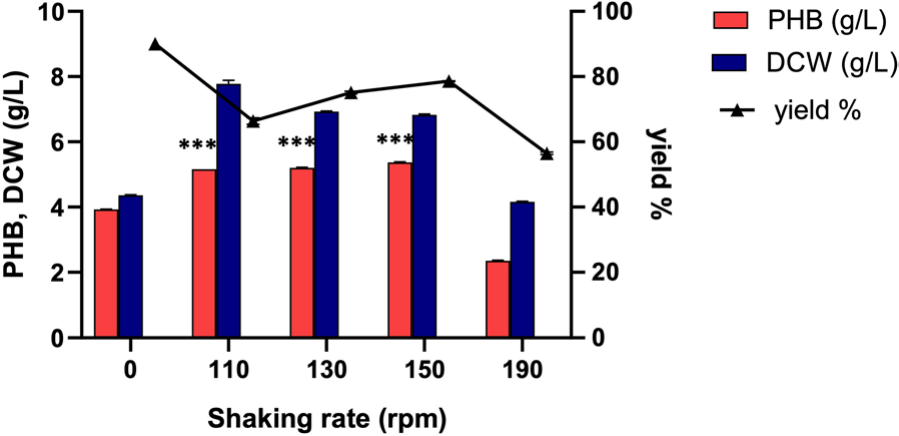
Effect of shaking rate on PHB production by the ES021 strain. Incubation temperature was 30°C for 48 h in the fermentation medium M4 at pH = 7 and an inoculum size of 1%. *** *P* < 0.001. Data are presented as values of three independent replicates. The error bars represent the standard error of the mean

To assess the impact of shaking rate on PHB production (Fig. 11 and Additional file 3: Table S4), fermentation experiments were conducted under both static and shaking conditions (110, 130, 150, and 190 rpm). Shaking the culture during fermentation proved to be advantageous for achieving higher PHB production levels. The ES021 strain exhibited a significant increase in PHB production under shaking conditions. Elevating the shaking rate from 110 to 150 rpm resulted in an increase in PHB production. These findings align with previous research demonstrating that increasing the shaking rate enhances PHB production in bacteria [63].

### Incubation period

**Fig. 12:**
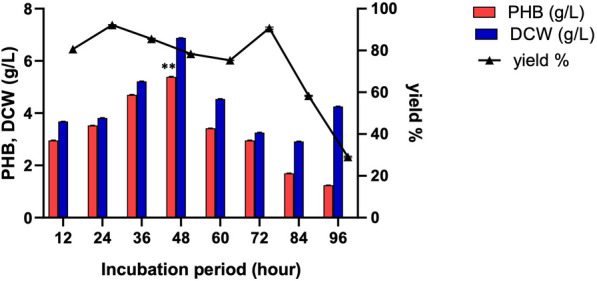
Effect of the incubation period on PHB production by the ES021 strain. Incubation temperature was at 30°C and shaking at 150 rpm in the fermentation medium M4 at pH = 7 and an inoculum size of 1%. **P value < 0.01. Data are presented as values of three independent replicates. The error bars represent the standard error of the mean

Figure12 and Additional file 3: Table S5 show the effect of various incubation periods on PHB production by the ES021 strain. The PHB production increased in a time dependent manner. A significantly higher amount of PHB (5.39 g/L) was obtained after 48 h. However, the production levels of PHB decreased to 2.95 and 1.21 after 72 and 96 h, respectively. Accordingly, these results revealed that the maximal PHB accumulation by the ES021 strain was at 48 h. Consistently, Bonartseva et al. showed that the observed maximal PHB accumulation by *Rhizobium* sp. was at 48 h [64]. This was also co-related entirely with previous studies showing that optimal time course for PHB production was at 48 h in *Pseudomonas* sp., *Bacillus* sp., and *Rhizobium alti* [12, 65].

### Production of PHB from molasses and a mixture of salted whey and molasses

Molasses, an economical industrial by-product compared to glucose, was used in this study as a carbon source by the ES021 strain for PHB synthesis (Table 5). PHB productivity exhibited a significant increase when utilizing molasses containing 5% sucrose (13.34 g/L with a PHB yield of 87.25%). Conversely, PHB production amount and yield decreased when employing a mixture of SW and molasses (10 and 15%). Belal reported PHB production from molasses ranging from 0.7 to 2.2 g/L at inoculum sizes of 1 and 2%, respectively [63]. Page et al. and Beaulieu et al. demonstrated the use of molasses as the sole carbon source to produce PHB by *Azotobacter vinelandii* and *Alcaligenes eutrophus*, respectively [66, 67]. Gouda et al. obtained a maximum PHB production with sugarcane molasses and glucose as sole carbon sources (40.8 and 39.9 per mg cell dry matter, respectively). The 3% molasses resulted in higher biomass, while the maximum yield of PHB (46.2% per mg cell dry matter) was achieved with 2% molasses [68].

**Table 5:** Effect of different sugar sources and concentrations on PHB production by the ES021 strain

Fermentation media	Concentration of sugars (%)	PHB g/L	DCW g/L	PHB yield
Molasses	5	13.34 ± 0.05****	15.29 ± 0.1	87.25
Molasses	10	6.63 ± 0.05	12.3 ± 0.03	53.9
SW + molasses 1:1	10	0.82 ± 0.01	6.98 ± 0.04	11.75
SW + molasses 2:1	15	1.1 ± 0.01	11.89 ± 0.02	9.25
LSD 0.05		0.1274	0.0282	

Incubation temperature was at 30 °C and shaking rate at 150 rpm, pH=7, incubation period of 48 h, and an inoculum size of 1%. **** *P* value <0.0001. Data are represented as means of three independent replicates ± standard error of the mean

### Scaling up PHB production in a 20 L bioreactor

To improve the PHB and biomass production of *salinicola salaries* ES021 strain, a batch fermentation was carried out in a 20 L stirred-tank bioreactor containing 15 L of the modulated medium (molasses 5%, NaCl 3%, yeast extract 4 g/L, MgSO_4_ 0.5 g/L, KH_2_PO_4_ 1 g/L). The temperature, pH, aeration, and agitation speed were maintained at 30°C, 7.0, 2.5 vvm, and 150 rpm, respectively. At 48 h of fermentation, PHB was accumulated by ES021 strain in an amount of 12.88 g/L, and the DCW was 25.12 g/L. Previous studies have demonstrated that the PHB production and biomass can be significantly enhanced in a 20 L stirred-tank bioreactor [69, 70].

### FTIR analysis

**Fig. 13:**
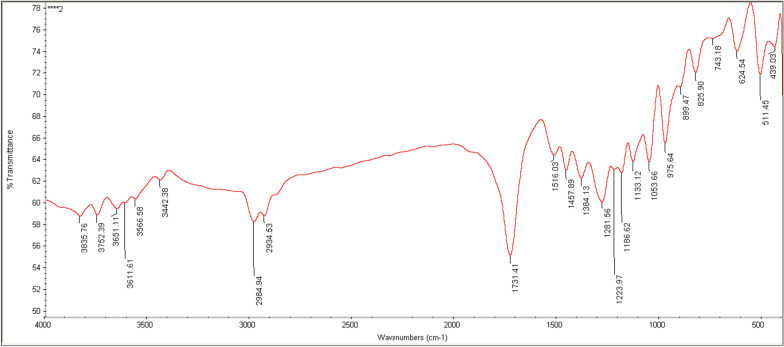
FTIR spectrum of PHB produced by the ES021 strain

The extracted polymer from the ES021 strain was used for determination of IR spectra. The FTIR spectrum of the produced PHB (Fig. 13) shows significant peaks at different wavelengths representing the PHB features. The FTIR spectrum of the produced polymer using sucrose as substrate was compared with the PHB standard and FTIR spectrum described by Brinda et al. [71]. The extracted polymer showed peaks at 3442, 2976 and 2934, 1731 and intense peaks at 1133 and 1053 cm^-1^. This indicates the presence of O–H stretching of alcohol, C–H stretching of alkanes, and C=O and C–O stretching of ester, respectively**.** Additionally, the range (899–511 cm^-1^) in the FTIR spectrum refers to the zone of carbon fingerprinting located in the PHB polymer [72].

### GC–MASS analysis

Table 6 shows the biodegradable compounds obtained from the produced polymer by the ES021 strain. GC–MS analysis of the PHB standard exhibited the twenty-three different biodegradable compounds existed in the produced polymer as shown in (Additional file 3: Table S6). The major detected compounds were 9-Octadecenoic acid (Z)-, methyl ester with a molecular weight of 296 in both standard and ES021 strain. The results were analyzed and compared with the standard and those described by Abdelrhman et al. and Okwuobi and Ogunjobiand and confirmed that the produced polymer is PHB [40, 73].

**Table 6:** The chemical composition of the PHB polymer produced by the ES021 strain as revealed by GC–MS analysis

S/N.	Retention time (minutes)	% Area	Compound name	Molecular weigh	Molecular formula
1	3.98	2.87	5,8,11,14-Eicosatetraenoic acid, phenylmethyl ester, (all-Z)-benzyl	394	C27H38O2
2	11.76	3.05	Nonadecane	268	C19H40
3	11.96	22.30	1-Dodecanamine, N,N-dimethyl	213	C14H31N
4	12.23	4.44	15-Methyltricyclo[6.5.2(13,1 4)0.0(7,15)]pentadeca-1,3,5,7,9,1 1,13-heptene	206	C16H14
4	12.65	2.16	Hexadecane, 5-butyl	282	C20H42
5	15.75	17.11	1-Tetradecanamine, N,N-dimethyl-	241	C16H35N
6	16.61	2.31	Tetradecane, 2,6,10-trimethyl	240	C17H36
7	18.64	1.84	Phthalic acid, butyl undecyl ester	376	C23H36O4
8	19.52	6.07	7,9-Di-tert-butyl-1-oxaspiro(4,5)dec a-6,9-diene-2,8-dione	276	C17H24O3
9	20.23	2.00	Tetradecane, 2,6,10-trimethyl	240	C17H36
10	22.38	24.13	9-Octadecenoic acid (Z)-, methyl ester	296	C19H36O2
11	22.56	6.68	N-Methyl-N-benzyltetradecanamine	225	C22H39N
12	22.90	1.91	Heptacosane	380	C27H56
13	28.65	2.72	1,2-Benzenedicarboxylic acid	390	C24H38O4

### NMR analysis

**Fig. 14:**
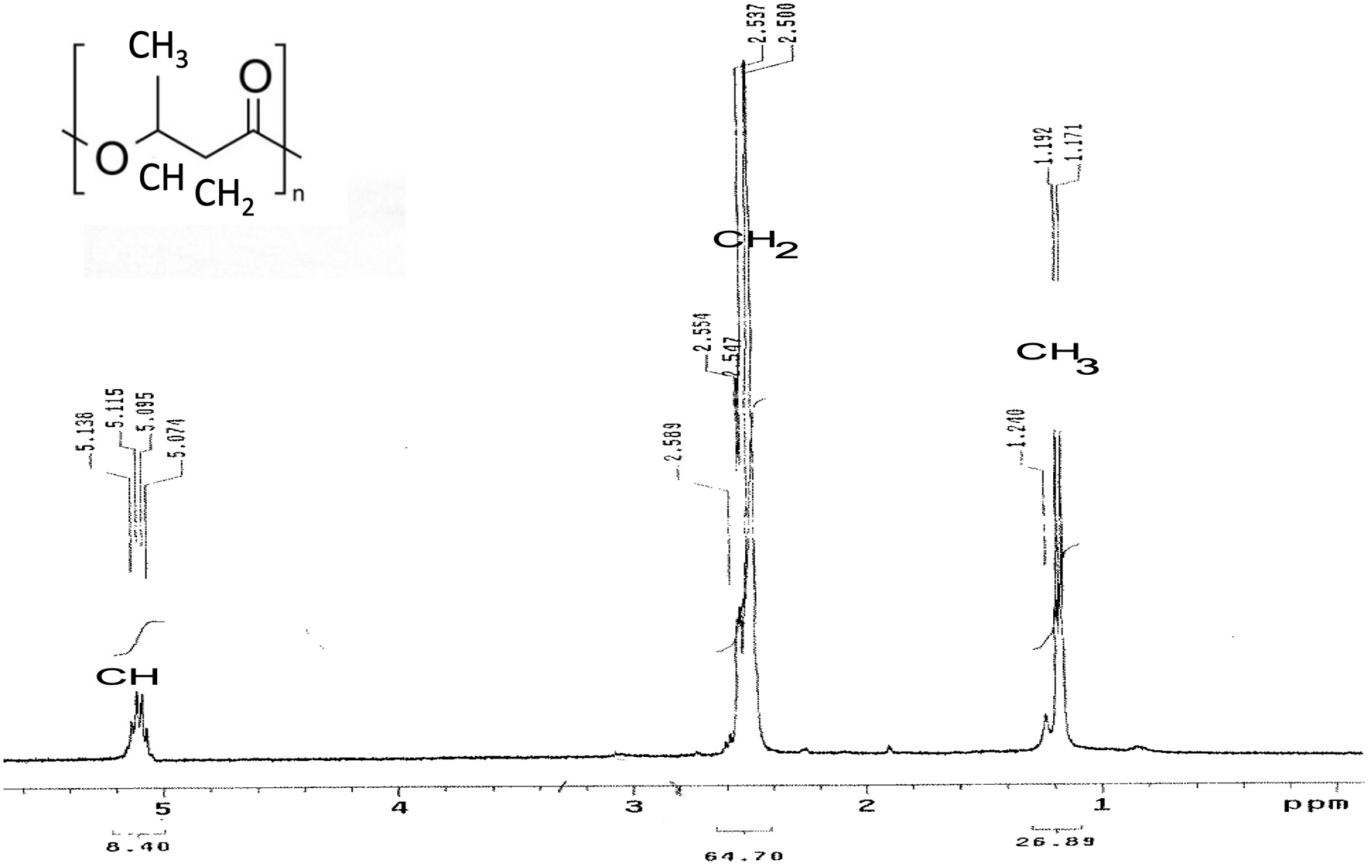
^1^H NMR spectrum of the PHB extracted from *Salinicola salarius* ES021 strain showing signals at chemical shift of δ = 1.17–1.23 ppm (CH_3_), 2.5–2.55 ppm (CH2), and 5.07–5.19 ppm (CH)

The ^1^H NMR spectrum of the produced PHB exhibited a doublet signal for a methyl group (–CH3) at a chemical shift of δ = 1.17–1.23 ppm. Signals arising from a methylene group attached to a carbonyl group (–CH2) were observed at a chemical shift of δ = 2.5–2.55 ppm. Multiple signals observed at a chemical shift of δ = 5.07–5.19 ppm were identified as originating from a methine group (-CH). All these signals collectively validate the structural composition of P(3-HB) (Fig. 14).

## Conclusions

*Salinicola salarius* ES021 strain is a potential and efficient natural-PHB producer from different carbon sources. The genome of the ES021 strain was characterized showing the genetic features and the putative PHB-related genes. *Salinicola salarius* ES021 strain has a unique genetic pathway for PHB production. However, further genetic engineering and construction of knockout mutants will be needed to confirm the involvement of the putative PHB-related genes in this strain. Optimization studies of the growth conditions were conducted in this study showing the effects of fermentation media, temperature, aeration, pH, inoculum size, and incubation period on the PHB production. *Salincola salarius* showed an enhanced PHB production on molasses. This indicates that *Salinicola salarius* ES021 strain can produce high amounts of PHB from different agro-industrial wastes such as salted whey and molasses.

### Supplementary Information

**Additional file 1. **The genetic distance between *Salinicola salarius* ES021 strain and other sequenced *Salinicola* species and other information related to other sequenced *Salinicola* species.

**Additional file 2. **The list of annotated genes, their functions, sequences, and locations in *Salinicola salarius* ES021 strain.

**Additional file 3: Table S1.** Effect of different incubation temperatures on PHB production by ES021. **Table S2.** Effect of different pH values on PHB production by ES021. **Table S3.** Effect of different inoculum sizes on PHB production by ES021. **Table S4.** Effect of different shaking rates on PHB production by ES021. **Table S5.** Effect of different incubation periods on PHB production by ES021. **Table S6.** GCMS analysis of PHB standard showing chemical composition of biodegradable polymer.

## Data Availability

The datasets generated or analyzed during this study are available and included in this publication (and its Additional files). The genomic raw sequence data are accessible with the following link https://www.ncbi.nlm.nih.gov/sra/PRJNA943367.

## Abbreviations

PHB: Poly(3-hydroxybutrate)
FTIR: Fourier-transform infrared spectroscopy
GC-MS: Gas chromatography-mass spectroscopy
PHAs: Polyhydroxyalkanoates
SW: Salted whey
SYA: Sucrose/Yeast extract agar
SYANa: Sucrose/Yeast extract agar medium supplemented with 3% Nacl
SBB: Sudan Black B
SYB: Sucrose/yeast extract broth
SYBNa: Sucrose/yeast extract broth medium supplemented with 3% NaCl
CDS: Protein-coding sequences
NCBI: National Center for Biotechnology Information
RASTtk: RAST tool kit
DCW: Dry cellweight
tRNA: Transfer RNA
rRNA: Ribosomal RNA
EC: Enzyme Commission
GO: Gene Ontology
MCL-PHA: Medium-chain-length PHA
EMP: Embden-Meyerhof-Parnas pathway
PP: Pentose phosphate pathway
ED: Entner-Doudoroff pathway
