# Supplementary material for: Genetic characterization of a novel Salinicola salarius isolate applied for the bioconversion of agro-industrial wastes into polyhydroxybutyrate
Source: Microb Cell Fact. 2024 Feb 17;23:56. doi: 10.1186/s12934-024-02326-z (PMC10874550; doi:10.1186/s12934-024-02326-z)
Supplement: Supplementary file 3 — Additional file 3: Table S1. Effect of different incubation temperatures on PHB production by ES021. Table S2. Effect of different pH values on PHB production by ES021. Table S3. Effect of different inoculum sizes on PHB production by ES021. Table S4. Effect of different shaking rates on PHB production by ES021. Table S5. Effect of different incubation periods on PHB production by ES021. Table S6. GCMS analysis of PHB standard showing chemical composition of biodegradable polymer. [file 12934_2024_2326_MOESM3_ESM.pdf]

Table S1. Effect of different incubation temperatures on PHB production by ES021

Temp	*PHB g/L*	*DCW g/L*	*PHB yield %*
25	1.54±0.02	5.99±0.01	25.71
27.5	2.02±0.05	5.10±0.06	39.55
30	3.46±0.02****	5.96±0.09	58.05
32.5	2.21±0.01	4.38±0.01	50.57
35	2.41± 0.06	2.82±0.01	85.46
LSD 0.05	**0.1358**	**0.6425**	

Incubation at 30°C for 48 h (110 rpm), inoculum size: 1%, fermentation medium: M4 with pH value of 7. **** P < 0.0001. Data are presented as values of three independent replicates. The error bars represent the standard error of the mean.

Table S2. Effect of different pH values on PHB production by ES021

pH	*PHB g/L*	*DCW g/L*	*PHB yield %*
6.2	3.46 ±0.02	5.96±0.01	58.05
6.5	3.74±0.01	5.96±0.01	84.09
7	5.16 ±0.01****	7.78±0.01	66.32
7.3	3.72±0.01	3.96±0.02	94.06
7.6	4.32 ±0.01	6.78±0.01	63.72
LSD 0.05	0.0461	0.3792	

Incubation at 30°C for 48 h (110 rpm), inoculum size: 1%, fermentation medium: M4. **** P < 0.0001. Data are presented as values of three independent replicates. The error bars represent the standard error of the mean.

Table S3. Effect of different inoculum sizes on PHB production by ES021

inoculum size	*PHB g/L*	*DCW g/L*	*PHB yield %*
0.5%	3.35±0.01	5.04±0.01	66.47
1%	5.16 ±0.01****	7.78±0.01	66.32
1.5%	3.72±0.02	1.72±0.01	34.69
2%	3.92 ±0.01	15.29± 0.01	25.64
2.5%	3.63±0.01	14.32±0.02	24.94
3%	3.38 ±0.04	16.71±0.01	20.23
LSD 0.05	0.0873	0.2365	

Incubation at 30°C for 48 h (110 rpm), fermentation medium: M4 with pH value of 7. **** $P < 0.0001$. Data are presented as values of three independent replicates. The error bars represent the standard error of the mean.

Table S4. Effect of different shaking rates on PHB production by ES021

Shaking rate	*PHB g/L*	*DCW g/L*	*PHB yield %*
Static	3.9 ±0.01	5.12± 0.02	76.17
110 rpm	5.16 ±0.01***	7.78±0.01	66.32
130 rpm	5.20±0.02***	6.13±0.02	75.14
150 rpm	5.39 ±0.02***	6.86±0.02	78.57
190 rpm	2.35±0.02	4.16±0.02	56.49
LSD 0.05	0.0524	0.2429	

Incubation at 30°C for 48 h, inoculum size: 1%, fermentation medium: M4 with pH value of 7. *** $P < 0.001$. Data are presented as values of three independent replicates. The error bars represent the standard error of the mean.

Table S5. Effect of different incubation periods on PHB production by ES021

incubation period	*PHB g/L*	*DCW g/L*	*PHB yield %*
12 h	2.96±0.01	3.68±0.01	80.60
24 h	3.53 ±0.01	3.82±0.01	92.24
36 h	4.70±0.02	5.27±0.01	85.58
48 h	5.39 ±0.02**	6.89±0.01	78.34
60 h	3.42±0.01	4.54±0.02	75.31
72 h	2.96± 0.01	3.26±0.01	90.71
84 h	1.70±0.01	2.92±0.01	58.27
96 h	1.24± 0.01	4.26±0.01	29.01
LSD 0.05	0.1274	0.0282	

Incubation at 30°C, inoculum size: 1%, fermentation medium: M4 with pH value of 7.
**$P < 0.01$. Data are presented as values of three independent replicates. The error bars represent the standard error of the mean.

Table S6. GC-MS analysis of PHB standard showing chemical composition of biodegradable polymer

S/N	Retention time (minutes)	% Area	Compound Name	Molecular Weight	Molecular Formula
**1**	3.95	1.71	Diallyl disulphide	146	$C_6H_{10}S_2$
**2**	4.31	0.91	Diallyl disulphide	146	$C_6H_{10}S_2$
**3**	7.49	0.75	Tetradecane, 2,6,10-trimethyl-	240	$C_{17}H_{36}$
**4**	7.85	0.69	Trisulfide, di-2-propenyl	178	$C_6H_{10}S_3$
**5**	8.01	4.67	Trisulfide, di-2-propenyl	178	$C_6H_{10}S_3$
**6**	8.18	0.87	Phenol, 2-methyl-5-(1methyl ethyl	150	$C_{10}H_{14}O$
**7**	9.28	1.03	5-Methyl-1,2,3,4-tetrathiane	170	$C_3H_6S_4$
**8**	11.76	1.09	Decane, 2,3,5,8-tetramethyl	198	$C_{14}H_{30}$
**9**	11.96	20.26	1-Dodecanamine, N,N-di methyl	213	$C_{14}H_{31}N$
**10**	12.22	3.08	3,4-Di hydro-2H-1,5-(3"-T-butyl) benzo dioxepine	206	$C_{13}H_{18}O_2$
**11**	12.65	0.68	Pentacosane	352	$C_{25}H_{52}$
**12**	13.65	0.58	1-Nonadecene	266	$C_{19}H_{38}$
**13**	15.75	8.72	Nizatidine	331	$C_{12}H_{21}N_5O_2S_2$
**14**	16.62	1.24	Tetradecane, 2,6,10-trimethyl	240	$C_{17}H_{36}$
**15**	17.33	0.50	1-Nonadecene	266	$C_{19}H_{38}$

**16**	19.53	1.72	7,9-Di-tert-butyl-1-oxaspiro(4,5)deca-6,9-diene-2,8-dione	276	$C_{17}H_{24}O_3$
**17**	19.61	23.13	Hexadecanoic acid, methyl ester	270	$C_{17}H_{34}O_2$
**18**	20.23	0.76	Tetradecane, 2,6,10-trimethyl-	240	$C_{17}H_{36}$
**19**	22.38	22.30	9-Octadecenoic acid (Z)-, methyl ester	296	$C_{19}H_{36}O_2$
**20**	22.55	3.38	2-Methyl enebrexane	134	$C_{10}H_{14}$
**21**	22.90	0.68	Docosane	310	$C_{22}H_{46}$
**22**	23.53	0.51	Dotriacntane	450	$C_{32}H_{66}$
**23**	25.60	0.75	N-Methyl-N-benzyl tetra decanamine	317	$C_{22}H_{39}N$
